# US National Trends in Pediatric Deaths From Prescription and Illicit Opioids, 1999-2016

**DOI:** 10.1001/jamanetworkopen.2018.6558

**Published:** 2018-12-28

**Authors:** Julie R. Gaither, Veronika Shabanova, John M. Leventhal

**Affiliations:** 1 Department of Pediatrics, Yale School of Medicine, New Haven, Connecticut

## Abstract

**Importance:**

It is not yet known how many children and adolescents die each year from opioid poisonings and how mortality rates have changed over time.

**Objective:**

To examine national trends in pediatric deaths from prescription and illicit opioids.

**Design, Setting, and Participants:**

Cross-sectional in which serial mortality data from the Centers for Disease Control and Prevention were analyzed. The population included 8986 children and adolescents (age, <20 years) who died in all US settings from opioid poisonings between 1999 and 2016. Data were collected and analyzed between June 1 and October 31, 2018.

**Exposures:**

All opioids.

**Main Outcomes and Measures:**

Age-specific mortality rates per 100 000 were estimated with smoothing spline Poisson regression.

**Results:**

Of the 8986 children and adolescents who died between 1999 and 2016 from prescription and illicit opioid poisonings, 6567 (73.1%) were male, 7921 (88.1%) were among adolescents aged 15 to 19 years, and 605 (6.7%) were among children aged 0 to 4 years. The overall pediatric mortality rate increased from 0.22 (95% CI, 0.19-0.25) to 0.81 (95% CI, 0.76-0.88) per 100 000, an increase of 268.2% (*P* for time effect <.001). The highest annual rates were among adolescents aged 15 to 19 years, but time trends revealed a steady linear increase among children aged 0 to 4 years and those aged 5 to 9 years as well as a steady linear increase until 2008 among the cohorts aged 10 to 14 years and 15 to 19 years, when rates briefly declined before rising again. Among adolescents aged 15 to 19 years, heroin was implicated in 1872 deaths: rates increased from 0.21 (95% CI, 0.17-0.25) to 1.06 (95% CI, 0.97-1.17), an increase of 404.8%, whereas rates for prescription opioids increased by 94.7% from 0.57 (95% CI, 0.49-0.66) to 1.11 (95% CI, 0.99-1.25) (all *P* for time effect <.001). Between 2014 and 2016, there were 1508 opioid deaths among adolescents aged 15 to 19 years; of these, 468 (31.0%) were attributed to synthetic opioids. Across time, 7263 (80.8%) of all pediatric deaths were unintentional, 5537 (61.6%) occurred outside of a medical facility, and 3419 (38.0%) children died at home or another residential setting. Among children younger than 5 years, 148 (24.5%) deaths were attributed to homicide.

**Conclusions and Relevance:**

Over 18 years, nearly 9000 children and adolescents died from opioid poisonings, and the mortality rate increased nearly 3-fold. These findings suggest that the opioid epidemic is likely to remain a growing public health problem in the young unless legislators, public health officials, clinicians, and parents take a wider view of the opioid crisis and implement protective measures that are pediatric specific and family centered.

## Key Points

**Question:**

How have US mortality rates for pediatric opioid poisonings changed over the past 2 decades?

**Findings:**

In this cross-sectional study, 8986 children and adolescents died between 1999 and 2016 from prescription and illicit opioid poisonings. During this time, the mortality rate increased 268.2%.

**Meaning:**

Pediatric-specific and family-centered interventions are needed to address pediatric opioid poisonings, a growing public health problem in the United States.

## Teaser

This cross-sectional study evaluates the changes in the mortality rates among US children and adolescents who died from prescription and illicit opioid poisonings between 1999 and 2016.

## Introduction

In 2015, there were 33 000 deaths in the United States attributed to opioid poisonings^1^; in 2016, deaths exceeded 43 000—more than for any other year on record.^2^ That Americans continue to die in unprecedented numbers from prescription opioids and, increasingly, heroin and illicitly manufactured fentanyl, despite aggressive public health measures to contain the crisis, speaks to the complexity and evolving nature of this epidemic.^2,3,4,5^

What began more than 2 decades ago as a public health problem primarily among young and middle-aged white males is now an epidemic of prescription and illicit opioid abuse that is taking a toll on all segments of US society, including the pediatric population. Millions of children and adolescents are now routinely exposed in their homes, schools, and communities to these potent and addictive drugs.^6,7,8,9,10,11,12,13^

Across the United States, nearly 5000 children younger than 6 years are evaluated annually in emergency departments for opioid exposures.^14^ In addition, hospitalizations for opioid poisonings increased nearly 2-fold across all pediatric age groups between 1997 and 2012.^15^ Rates more than doubled among children aged 1 to 4 years, and in adolescents aged 15 to 19 years, poisonings attributed to suicidal and unintentional intent increased 2- and 3-fold, respectively.

It is unclear, however, how many children die each year in the United States from opioid poisonings and how mortality rates have changed over time since the epidemic began in the late 1990s.^10,16,17,18^ The objective of this study, therefore, was to examine in detail national trends in pediatric deaths from opioid poisonings in association with age, race/ethnicity, manner of death (ie, intent), implicated opioid, and setting (eg, medical vs residential).

## Methods

### Study Overview

We examined national trends in pediatric deaths from opioid poisonings stratified by age, race/ethnicity, manner of death, type of opioid, and setting. This study followed the Strengthening the Reporting of Observational Studies in Epidemiology (STROBE) reporting guideline.^19^ This research involved deidentified data and was considered exempt from approval by the institutional review board of the Yale School of Medicine.

### Study Design and Data Source

We conducted a retrospective analysis of serial cross-sectional data available from the Centers for Disease Control and Prevention (CDC) Wide-Ranging Online Data for Epidemiologic Research (WONDER)^20^ database, which compiles county-level mortality data from all US death certificates from the National Center for Health Statistics.

### Identification of Opioid Deaths

We used the Multiple Cause of Death file^21^ within CDC WONDER to identify poisonings from prescription and illicit opioids that occurred between January 1, 1999, and December 31, 2016. Deaths from any opioid (prescription or illicit) were identified using the following *International Statistical Classification of Diseases, Tenth Revision (ICD-10)*^22^ codes: T40.0 (opium), T40.1 (heroin), T40.2 (natural and semisynthetic opioids), T40.3 (methadone), T40.4 (synthetic opioids excluding methadone, which we refer to throughout as *synthetic opioids*), and T40.6 (other and unspecified narcotics). We grouped all codes other than those for opium, heroin, and synthetic opioids under the label *prescription opioids*. Synthetic opioids were excluded from this category owing to the recent surge in deaths from illicitly manufactured fentanyl,^23^ which cannot be distinguished from pharmaceutical fentanyl under the current T40.4 code.^24^ Opium, for which there was only 1 death with this code, was not categorized as either a prescription or illicit opioid. We included methadone in all analyses of prescription opioids; however, we singled out this drug for a separate analysis as a means to understand the plateau in overall mortality rates between 2012 and 2014 (further details are provided in the Discussion section).

We then classified deaths according to intent using the following *ICD-10* underlying cause-of-death codes: X40-44 (unintentional), X60-64 (suicide), X85 (homicide), and Y10-Y14 (undetermined intent). The United States has used *ICD-10* coding on death certificates since 1999.^25^

### Identification of Pediatric Deaths

We limited the sample to those younger than 20 years, and, to be consistent with the age stratifications used by the CDC,^20,26^ we categorized children and adolescents by the following ages: 0 to 4, 5 to 9, 10 to 14, and 15 to 19 years. Only 26 children and adolescents younger than 15 years died from heroin, and 129 died from synthetic opioids; therefore, we restricted these 2 analyses to adolescents aged 15 to 19 years.

### Identification of Coingestions for Other Prescription and Illicit Substances

In the oldest age group, we also examined deaths involving 1 or more prescription or illicit substances using the relevant *ICD-10* codes. The agents included benzodiazepines (T42.4), cocaine (T40.5), alcohol (T51.x), antidepressants (T43.0-T43.2), psychostimulants (T43.6), cannabis (T40.7), antipsychotics/neuroleptics (T43.3-T43.5), and barbiturates (T43.2).

### Statistical Analysis

Data were collected and analyzed between June 1 and October 31, 2018. Descriptive statistics were used to characterize the sample, and differences in demographic and clinical characteristics were assessed with χ^2^ tests.

We used a generalized smoothing spline Poisson regression model to estimate mortality rates and assess temporal changes in rates over time (ie, time effect).^27,28,29^ This robust, nonparametric approach accounts for the random error inherent in mortality rates caused by fluctuations in the number of deaths across time.^30^ In this model, rates were represented by the Poisson distribution, with the natural log of those at risk as the offset variable, and were modeled with cubic splines. The number of knots was determined by the *max(30,10N^[2/9]^)* specification, where N equaled the sample size.^27,31^

Data smoothing was used in this analysis to address the unreliability of the crude mortality estimates that were based on small death counts. Smoothing parameters were determined by cross-validation,^32^ and the penalized likelihood method was used to obtain point estimates and compute approximate 95% Bayesian CIs.^33^ Further details on cross-validation and the use of smoothing techniques to improve the reliability of demographic data with small cell sizes (eg, n <10) are available elsewhere.^28,29,32,34^

Descriptive analyses were performed using SAS software, version 9.4 (SAS Institute Inc); smoothing spline Poisson regression models were evaluated in R, version 3.5.1 (R Core Team, University of Auckland), with R Package gss.^31^ A 2-sided statistical significance level of .05 was applied to all analyses.

## Results

### Demographic Characteristics

A total of 8986 children and adolescents died from prescription and illicit opioid poisonings between 1999 and 2016. As reported in the Table, 7921 (88.1%) deaths were among adolescents aged 15 to 19 years, and 605 (6.7%) were among children aged 0 to 4 years. Of all deaths, 7183 (79.9%) occurred in non-Hispanic white children and adolescents and 6567 (73.1%) in males.

**Table.** Pediatric Deaths From Prescription and Illicit Opioids, 1999-2016

Demographic and Clinical Characteristics	No. (%)
No.	8986
Age category, y	
0-4	605 (6.7)
5-9	96 (1.1)
10-14	364 (4.1)
15-19	7921 (88.1)
Sex	
Male	6567 (73.1)
Female	2419 (26.9)
Race	
Non-Hispanic white	7183 (79.9)
Non-Hispanic black	642 (7.1)
Hispanic	929 (10.3)
Other	232 (2.6)
Place of death	
Home	3419 (38.0)
Inpatient	939 (10.4)
Emergency department or outpatient	2165 (24.1)
Dead on arrival	345 (3.8)
Other or unknown	2118 (23.6)
Manner of death	
Unintentional	7263 (80.8)
Suicide	445 (5.0)
Homicide	219 (2.4)
Undetermined	1059 (11.8)

### Annual Death Counts

**Figure 1.**
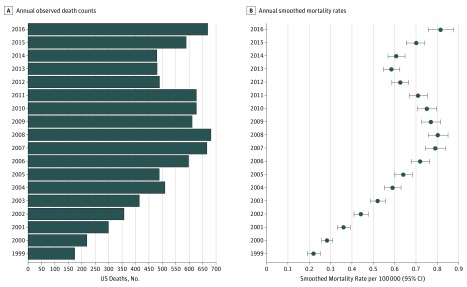
Number of Pediatric Opioid Deaths and Mortality Rates by Year Number of deaths (A) and mortality rates (B) for children and adolescents ages 0 to 19 years. Error bars indicate 95% CIs.

Figure 1 shows the annual number of deaths for the group overall. When stratified by age (eFigure in the Supplement and the Table), we found the following total number of deaths per age groups: 0 to 4 years, n = 605; 5 to 9 years, n = 96; 10 to 14 years, n = 364; and 15 to 19 years, n = 7921.

### Mortality Rates

**Figure 2.**
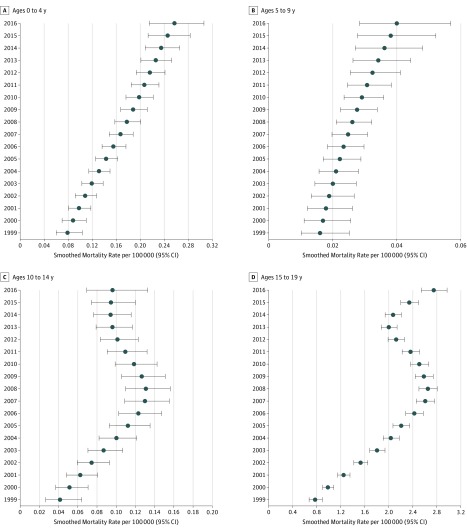
Age-Stratified Pediatric Mortality Rates by Year Deaths in children aged 0 to 4 years (A), 5 to 9 years (B), 10 to 14 years (C), and 15 to 19 years (D). Error bars indicate 95% CIs.

As shown in Figure 1, from 1999 to 2016, the annual estimated mortality rate for all children and adolescents rose from 0.22 (95% CI, 0.19-0.25) to 0.81 (95% CI, 0.76-0.88) per 100 000, an increase of 268.2% (*P* for time effect <.001). Figure 2 shows temporal trends in mortality rates when stratified by the 4 age categories. For children aged 0 to 4 years, rates increased from 0.08 (95% CI, 0.06-0.10) in 1999 to 0.26 (95% CI, 0.22-0.31) in 2016, an increase of 225.0% (*P* for time effect <.001). Among children aged 5 to 9 years, rates rose from 0.02 (95% CI, 0.01-0.03) to 0.04 (95% CI, 0.03-0.06), an increase of 100.0%, and for those aged 10 to 14 years, rates rose from 0.04 (95% CI, 0.03-0.06) to 0.10 (95% CI, 0.07-0.13), an increase of 150.0% (all *P* for time effect <.001). Adolescents aged 15 to 19 years had the highest annual rates for each of the 18 years examined; in this group, rates increased from 0.78 (95% CI, 0.68-0.88) in 1999 to 2.75 (95% CI, 2.55-2.96) in 2016, an increase of 252.6% (*P* for time effect <.001).

**Figure 3.**
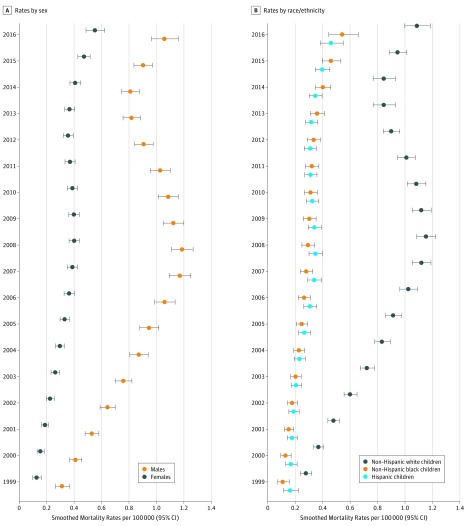
Pediatric Mortality Rates by Year Stratified by Sex and Race/Ethnicity for Children and Adolescents Aged 0 to 19 Years Deaths shown by sex (A) and race/ethnicity (B). Error bars indicate 95% CIs.

As shown in Figure 3, the overall pediatric mortality rate for males (n = 6567) increased from 0.31 (95% CI, 0.27-0.36) to 1.06 (95% CI, 0.97-1.15), an increase of 241.9%, compared with females (n = 2419), in whom rates increased by 323.1% from 0.13 (95% CI, 0.10-0.15) to 0.55 (95% CI, 0.49-0.62) (all *P* for time effect <.001).

Also shown in Figure 3 are mortality rates according to race/ethnicity. For non-Hispanic white children and adolescents (n = 7183), rates increased from 0.28 (95% CI, 0.24-0.32) to 1.09 (95% CI, 1.00-1.18), an increase of 289.3% (*P* for time effect <.001). Comparatively, for non-Hispanic black children and adolescents (n = 642), rates increased by 390.9% from 0.11 (95% CI, 0.08-0.15) to 0.54 (95% CI, 0.45-0.65), whereas for Hispanic children and adolescents (n = 929), rates increased by 187.5% from 0.16 (95% CI, 0.12-0.22) to 0.46 (95% CI, 0.39-0.55) (all *P* for time effect <.001).

### Manner of Death

Among all children and adolescents, 7263 deaths (80.8%) were unintentional, while 445 (5.0%) and 219 (2.4%) were attributed to suicide and homicide, respectively (Table). Manner of death varied significantly when examined by age group (*P* < .001). Among those aged 15 to 19 years, 6755 (85.3%) of deaths were unintentional, while 381 (4.8%) deaths were attributed to suicide. Among children younger than 5 years, 230 (38.0%) deaths were unintentional, 148 (24.5%) were due to homicide, and the manner of death could not be determined in 227 (37.5%) of the cases; the percentage of deaths due to homicide was highest for those younger than 1 year at 34.5% (n = 57).

### Type of Opioid

Prescription opioids were implicated in 6561 deaths (73.0%), and the mortality rate increased by 131.3% from 0.16 (95% CI, 0.14-0.19) to 0.37 (95% CI, 0.33-0.41) (*P* for time effect <.001); methadone alone was implicated in 2358 (35.9%) prescription opioid deaths. However, the mortality rate for methadone peaked at 0.30 (95% CI, 0.28- 0.33) in 2007 and by 2016 had decreased to 0.07 (95% CI, 0.06-0.09), a decrease of 76.7% (*P* for time effect <.001).

**Figure 4.**
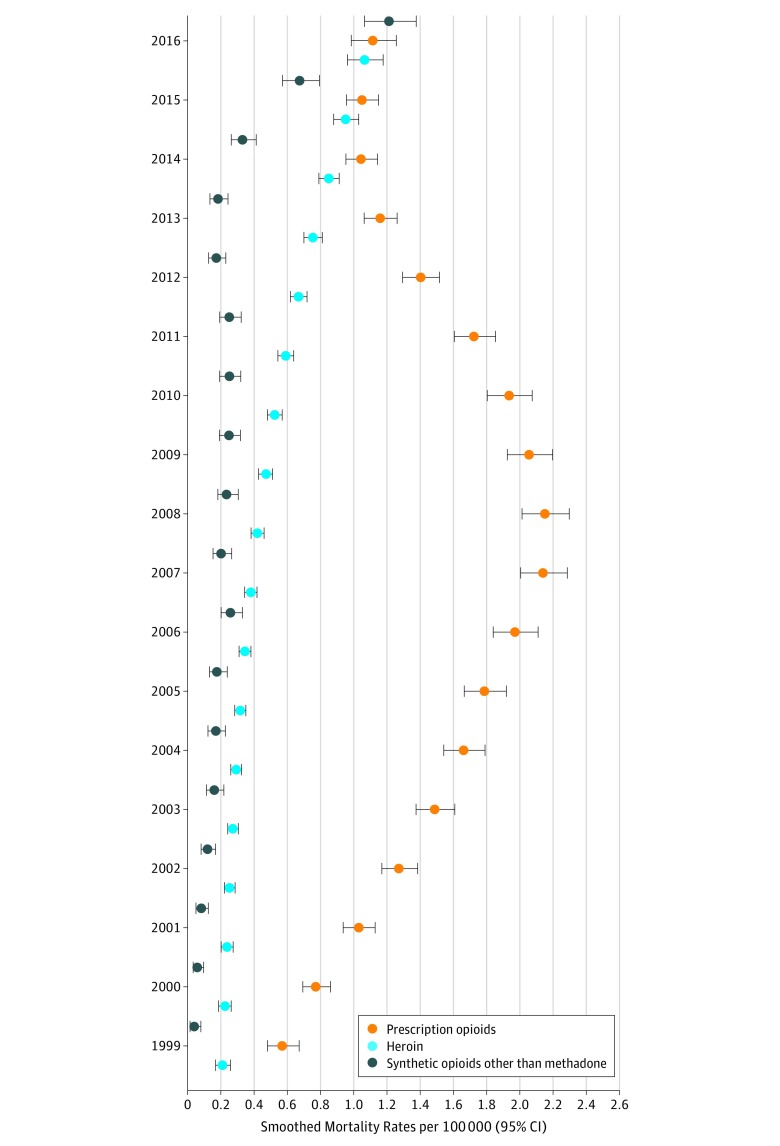
Pediatric Mortality Rates by Year Stratified by Type of Opioid for Adolescents Aged 15 to 19 Years Error bars indicate 95% CIs.

Among adolescents aged 15 to 19 years, heroin was implicated in 1872 deaths (23.6%). As shown in Figure 4, rates for fatal heroin poisonings in this group increased from 0.21 (95% CI, 0.17-0.25) to 1.06 (95% CI, 0.97-1.17), an increase of 404.8%, whereas rates for prescription opioids increased by 94.7% from 0.57 (95% CI, 0.49-0.66) to 1.11 (95% CI, 0.99-1.25) (all *P* for time effect <.001). In this age group, mortality rates for synthetic opioids increased from 0.04 (95% CI, 0.02-0.07) to 1.21 (95% CI, 1.07-1.37), an increase of 2925.0% (*P* for time effect <.001). Of the 1023 deaths attributed to these drugs between 1999 and 2016, 468 (45.7%) occurred between 2014 and 2016. During these 3 years, there were 1508 opioid deaths among adolescents aged 15 to 19 years: of these, 468 (31.0%) were attributed to synthetic opioids.

### Coingestions

Among adolescents aged 15 to 19 years, we found that 3050 deaths (38.5%) also involved 1 or more prescription or illicit substances in addition to an opioid. These included benzodiazepines, 1553 (19.6%); cocaine, 919 (11.6%); alcohol, 523 (6.6%); antidepressants, 325 (4.1%); psychostimulants, 317 (4.0%); cannabis, 127 (1.6%); antipsychotics/neuroleptics, 95 (1.2%); or barbiturates, 24 (0.3%).

### Place of Death

A total of 3419 deaths (38.0%) occurred in the home or another residential setting. In all, 5537 deaths (61.6%) occurred outside of a medical facility; only 939 (10.4%) children and adolescents died as inpatients, and 2510 (27.9%) died either in the emergency department, another outpatient setting, or were dead on arrival, which the CDC counts among medical facility deaths.^21^

## Discussion

To our knowledge, this is the first study to examine national data on deaths in children and adolescents from prescription and illicit opioid poisonings. Nearly all of what is currently known about the epidemiology of fatal opioid poisonings in the United States comes from the adult overdose literature, where it is common to either exclude deaths in the young from the analysis or group them into 1 (eg, age, <25 years) or 2 (eg, age 0-14 and 15-24 years) broad categories.^1,2,3,35^ By aggregating deaths in infants, children, adolescents, and young adults together, the extent to which the pediatric population has been harmed by what is increasingly a systemic crisis is obscured.

In this study of age-specific mortality rates, we found that, between 1999 and 2016, nearly 9000 children and adolescents died from prescription and illicit opioid poisonings in the United States, and the pediatric mortality rate for these drugs increased nearly 3-fold. Annual rates were highest for adolescents aged 15 to 19 years, but across all age groups, we found a substantial rise in rates across time. The largest changes were seen among the oldest and youngest children: rates increased by 252.6% among adolescents aged 15 to 19 years, followed by a 225.0% increase among children aged 0 to 4 years. In children aged 5 to 9 and 10 to 14 years, rates increased by 100.0% and 150.0%, respectively.

The majority of deaths were among non-Hispanic white males, but with each passing year, non-Hispanic black children accounted for a larger proportion of fatalities; compared with an almost 3-fold increase among white children, black children had a near 4-fold increase. A similar trend was seen for females, in whom rates increased more than 3-fold, compared with a 2-fold increase among males. These shifting demographic trends mirror those seen in the adult population, where mortality rates are rising rapidly among those of black race and women.^2,36^

Most of the deaths were unintentional, with only 5.0% and 2.4% attributed to suicide and homicide, respectively. Even among older adolescents, the pediatric age group with the highest suicide risk,^37^ 85.3% of the deaths were unintentional. In contrast, nearly 1 in 4 deaths (24.5%) were attributed to homicide among children younger than 5 years, and only 38.0% of deaths in this age group were clearly unintentional. In infants, 34.5% of deaths were considered homicides.

Across all age groups, the majority of deaths occurred outside of any medical setting: only 10.4% of deaths were in the inpatient setting and 24.1% were in the emergency department. A high percentage of children and adolescents (38.0%) died at home.

Most of the current studies that comment on pediatric deaths from opioids do so in the context of emergency department visits or hospitalizations. Results from this research have likely contributed to the perception that opioid poisonings rarely result in fatalities for children and adolescents. For example, a recent study of national trends in pediatric hospitalizations for prescription and illicit opioid poisonings found that, although approximately 2000 children a year, on average, were hospitalized between 1997 and 2012, only a small number died during hospitalization—approximately 30 per year.^15^ Results from the present study make it clear that the number of children and adolescents dying each year in the United States from opioid poisonings is actually closer to 500.

In general, pediatric mortality rates for opioid poisonings differ from those of adults in degree of magnitude, but both follow similar temporal and drug use patterns.^4,38,39,40,41,42^ We found, for instance, a steady linear increase in annual pediatric mortality rates until roughly 2008. Rates then began to decline and were essentially flat between 2012 and 2014 before they began rising again through 2016. The dip seen here corresponds with the decline in prescription opioid deaths noted by Dart et al,^43^ who found that mortality rates for the US population as a whole plateaued between 2011 and 2013. Similarly, Gaither et al^15^ showed that hospitalizations in children and adolescents for prescription opioid poisonings decreased from 2009 to 2012. The trends seen in both studies likely reflect the slight decrease in the number of opioid prescriptions dispensed during the years in question as well as the effect of numerous public health measures enacted to contain the epidemic.^43^

Research in subsequent years, however, has shown that any public health gains achieved in terms of reducing fatal and nonfatal poisonings from prescription opioids over the past decade have not been sustained in the wake of the rise in poisonings from heroin and, since 2014, illicitly manufactured fentanyl.^5,38,40^ We found in the present study that heroin was responsible for nearly 1 in 4 deaths (23.6%) among adolescents aged 15 to 19 years, and whereas mortality rates for prescription opioid poisonings increased 95% over time, rates for fatal heroin overdoses increased 404.8%. Deaths from synthetic opioids in this age group increased by 2925.0%; almost half of these deaths occurred in the latter 3 years of the study. Between 2014 and 2016, synthetic opioids were implicated in nearly one-third of all prescription and illicit opioid deaths among older teens.

The underrecognition of the risks that prescription and illicit opioids pose to children and adolescents is reflected in the current policies and practices in place in the United States today. Of the hundreds of state and federal initiatives enacted to contain the opioid crisis, nearly all focus on adults.

Of particular note is the lack of childproof packaging for many commonly prescribed opioids, including long-acting forms of the medications that present a known risk to children—especially small children—in whom even a minimal exposure can lead to respiratory arrest and death. For example, both Suboxone (the combination form of buprenorphine and naloxone), a medication used to treat opioid addiction, and Duragesic (the transdermal pain patch of fentanyl) come in foil wrappers that can be easily opened by a child. Suboxone is no longer sold in pill form because of concerns over pediatric exposures, but in its current formulation—which consists of brightly colored film strips—it still poses a danger to children.^44,45,46,47^

These risks are particularly relevant given that the field of medicine is moving into an era in which adults and adolescents with opioid use disorders are increasingly receiving medication-assisted treatment.^48^ While medication-assisted treatment is important and has received the endorsement of the American Academy of Pediatrics,^48^ pediatric exposures to methadone and buprenorphine are likely to increase in the coming years unless further safeguards are put into place. We found that methadone was implicated in more than one-third of all prescription opioid deaths. However, the mortality rate for methadone peaked in 2007 and declined steadily thereafter. This decline corresponds with a 2006 Public Health Advisory issued by the US Food and Drug Administration warning clinicians of the dangers of using methadone for pain management.^49,50^

In contrast, poisonings from buprenorphine have been increasing in recent years^51^; between 2007 and 2016, more than 11 000 calls were made to US poison control centers for pediatric buprenorphine exposures, for which nearly 90% were among children younger than 6 years, and the majority occurred in the child’s home.^52^ Other research has shown that for young children, nearly all prescription opioid poisonings occur when the child is exposed to a drug prescribed for a parent or other adult in the household.^6^

The nature of unintentional poisonings in young children as well as the substantial rise in adolescent deaths from heroin and synthetic opioids are particularly relevant to recent initiatives that would increase the availability of naloxone in homes and communities, especially given that the majority of pediatric deaths occur outside of a medical setting.^39,53^ Further study, however, is warranted to determine what risks and benefits such policy changes would have on children and adolescents.

It is important to address the deaths seen in the youngest children (age, 0-4 years), a highly vulnerable group for which the consequences of the opioid crisis has been somewhat overshadowed by opioid-related morbidity among neonates and older teens.^54,55,56^ We found that children aged 0 to 4 years had the second-highest mortality rates overall as well as the second-largest increase in rates over time. A large percentage of the deaths were due to homicides and intentional harm. Further research is needed to determine what roles abuse, neglect, and parental substance abuse—specifically, opioid abuse—play in these deaths.

### Limitations

There are at least 2 primary limitations to this research. First, this study relies on population-based data collected from US death certificates. The quality of this information depends on the accuracy and completeness of the investigations conducted at the time of death.^57^ Therefore, as with all mortality data, there is the potential for misclassification of cause and manner of death. Second, we were unable to describe the circumstances behind the deaths or report precisely on the opioids implicated in the poisonings, including pharmaceutical vs illicitly manufactured fentanyl; thus, our analysis of prescription opioids, which excludes all synthetics other than methadone, underestimates deaths from prescribed medications.^23^

Despite its limitations, this study supports initiatives that would bring about much-needed changes to current policies, practices, and consumer product regulations. Such changes would reduce the harm done to children, families, and communities by these potent and addictive medications.

## Conclusions

The pediatric mortality rate for opioid poisonings increased nearly 3-fold in the United States between 1999 and 2016, and opioids were responsible for the deaths of 8986 children and adolescents. This public health problem is likely to grow unless parents, legislators, public health officials, and clinicians—including physicians who prescribe opioids to adults—begin to take a wider view of what is a systemic crisis. Isolated solutions that fail to account for how entire families and communities are affected by adult opioid use are unlikely to lead to a substantive reduction in opioid deaths for either children or adults.

## Notes

Supplement. **eFigure.** Age-Stratified Pediatric Death Counts
