# Supplementary material for: US National Trends in Pediatric Deaths From Prescription and Illicit Opioids, 1999-2016
Source: JAMA Netw Open. 2018 Dec 28;1(8):e186558. doi: 10.1001/jamanetworkopen.2018.6558 (PMC6324338; doi:10.1001/jamanetworkopen.2018.6558)
Supplement: Supplement. — eFigure. Age-Stratified Pediatric Death Counts [file jamanetwopen-1-e186558-s001.pdf]

Supplementary Online Content

Gaither JR, Shabanova V, Leventhal JM. US national trends in pediatric deaths from prescription and illicit opioids, 1999-2016. *JAMA Netw Open*. 2018;1(8):e186558.
doi:10.1001/jamanetworkopen.2018.6558

eFigure. Age-Stratified Pediatric Death Counts

This supplementary material has been provided by the authors to give readers additional information about their work.

© 2018 Gaither JR et al. *JAMA Network Open.*

eFigure. Age-Stratified Pediatric Death Counts

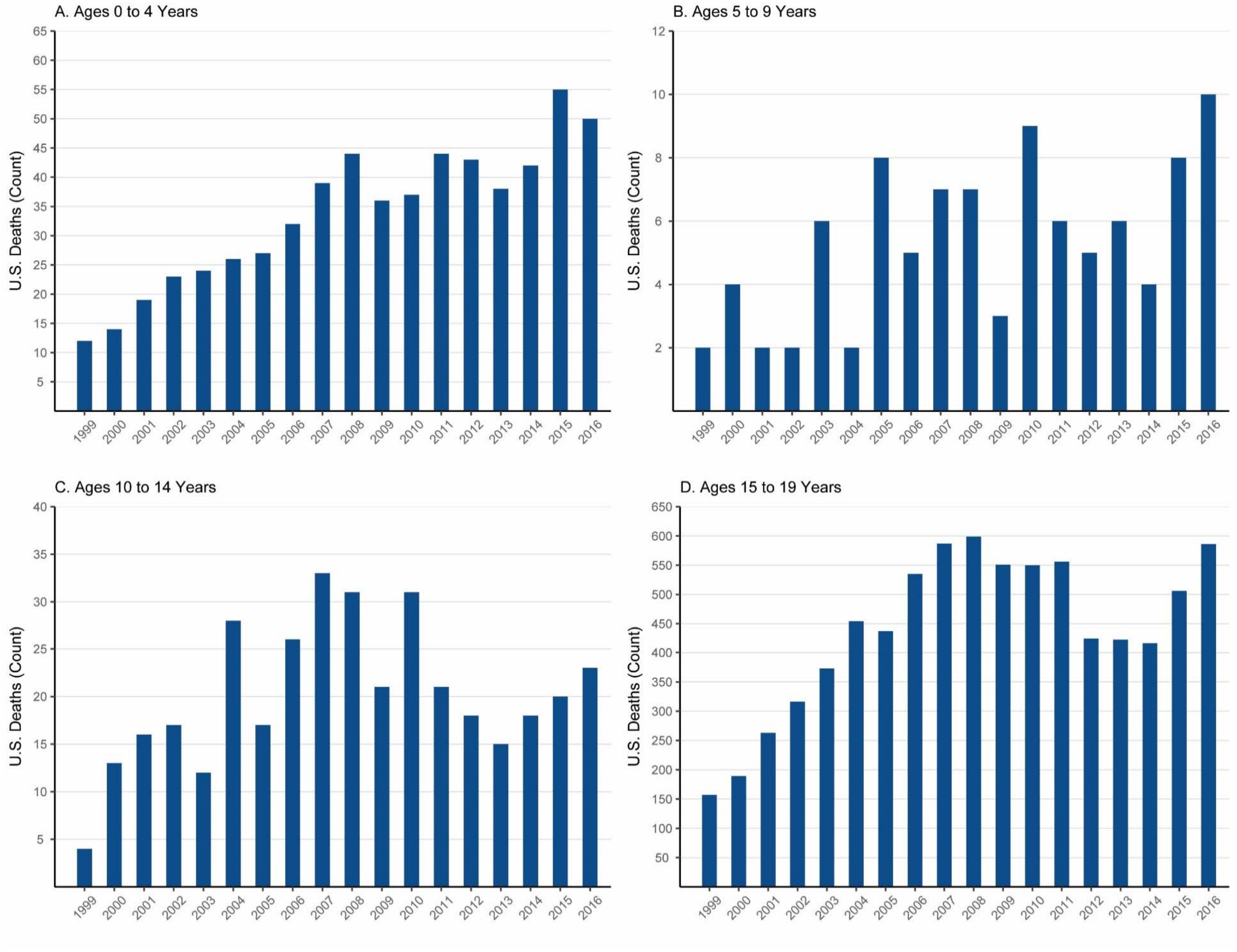

© 2018 Gaither JR et al. *JAMA Network Open.*
